# Gender-based reciprocal expression of transforming growth factor-β1 and the inducible nitric oxide synthase in a rat model of cyclophosphamide-induced cystitis

**DOI:** 10.1186/1476-9255-6-23

**Published:** 2009-08-19

**Authors:** Pradeep Tyagi, Vikas Tyagi, Naoki Yoshimura, Erich Witteemer, Derek Barclay, Patricia A Loughran, Ruben Zamora, Yoram Vodovotz

**Affiliations:** 1 Department of Urology, William Beaumont Hospital, MI 48073, USA; 2 Department of Urology, University of Pittsburgh, PA 15213, USA; 3 Department of Surgery, University of Pittsburgh, PA 15213, USA; 4 Center for Inflammation and Regenerative Modeling, McGowan Institute for Regenerative Medicine, University of Pittsburgh, PA 15219, USA

## Abstract

**Background:**

The pluripotent cytokine transforming growth factor-β1 (TGF-β1) is the central regulator of inducible Nitric Oxide Synthase (iNOS) that is responsible for nitric oxide (NO) production in inflammatory settings. Previous studies have implicated a role for NO, presumably derived from iNOS, in cyclophosphamide (CYP)-induced cystitis in the bladder. TGF-β1 is produced in latent form and requires dissociation from the latency-associated peptide (LAP) to act as primary anti-inflammatory and pro-healing modulator following tissue injury in the upper urinary tract. Since the role of TGF-β1 in lower urinary tract inflammation is currently unknown, and since gender-based differences exist in the setting of interstitial cystitis (IC), the present study examined the relationship between TGF-β1 and iNOS/NO in the pathogenesis of CYP-induced cystitis in both male and female rats.

**Methods:**

Sprague-Dawley rats, 4 months of age, of either gender were given 150 mg/kg CYP intraperitoneally. Urinary and bladder tissue TGF-β1 and NO reaction products (NO_2_^-^/NO_3_^-^) were quantified as a function of time following CYP. Expression of active and latent TGF-β1 as well as iNOS in harvested bladder tissue was assessed by immunohistochemistry.

**Results:**

Female rats had significantly higher levels of NO_2_^-^/NO_3_^- ^in urine even at baseline as compared to male rats (p < 0.001), whereas there was no gender based significant difference in urine levels of active or latent TGF-β1 prior to CYP injection. Inflammatory and cytotoxic changes were induced by CYP in the bladder of both sexes that were accompanied by differences in the urine levels of NO_2_^-^/NO_3_^- ^and TGF-β1. Male rats responded to CYP with significantly lower levels of NO_2_^-^/NO_3_^- ^and significantly higher levels of TGF-β1 in urine (p < 0.05) as compared to females at all time points after CYP. The urine levels of NO_2_^-^/NO_3_^- ^after CYP were inversely correlated to latent and active TGF-β1 (Pearson coefficient of -0.72 and -0.69 in females and -0.89 and -0.76 in males, respectively; p < 0.01). Bladder tissue of male rats exhibited significantly higher levels of both latent and active TGF-β1 (p < 0.01) compared to female rats after CYP. TGF-β1 and iNOS protein was mostly localized in the urothelium.

**Conclusion:**

The results of this study suggest that there exists an inverse relationship between the expression of TGF-β1 and iNOS/NO_2_^-^/NO_3_^- ^in CYP-inflamed bladder. The gender of the animal appears to magnify the differences in urine levels of TGF-β1 and NO_2_^-^/NO_3_^- ^in this inflammatory setting. These results support the hypothesis that TGF-β1 can suppress iNOS expression associated with bladder inflammation and reduce systemic levels of NO_2_^-^/NO_3_^-^, and further suggest that this feature of TGF-β1 can be harnessed for therapy and diagnosis of interstitial cystitis.

## Background

Cyclophosphamide is an oxazaphosphorine DNA alkylating agent, known for its anti-neoplastic and immunosuppressant properties, that is used clinically for malignancy, bone marrow transplantation, and multiple sclerosis. A prominent side effect of CYP is hemorrhagic cystitis [1,2]. It has been proposed that acrolein, a phase I metabolic product of CYP, is the causative agent of the edema, ulceration, and hemorrhage evident upon direct contact with bladder lumen [3]. This ability of CYP to cause cystitis has been utilized to simulate interstitial cystitis (IC) in pre-clinical studies [4].

A recent study from our laboratory suggested that changes in the cytokine milieu of the bladder after CYP describes a pro-inflammatory phenotype in this organ, likely due to the rapid infiltration of innate immune cells. These inflammatory changes correlate with the abnormal voiding and histology characteristic of cyclophosphamide (CYP)-induced cystitis in rats [4]. Temporal changes in the levels of pro-inflammatory cytokines and chemokines such as interleukin IL-1α, IL-1β, IL-6, IL-17, IL-18, and GRO/KC preceded or concurred with pathological changes induced by CYP. Studies from other groups demonstrate that various inflammatory cytokines seem to mediate the pathogenesis of CYP-induced cystitis through the induction of high levels of iNOS and NO production as well as cyclooxygenase-derived prostaglandins [5-8]. Clinical studies based on tissue biopsies from patients with IC suggest an elevated expression of both iNOS and TGF-β1 in the urothelium as compared to patients with kidney stone or benign hematuria [9,10].

TGF-β1 is expressed by inflammatory cells such as neutrophils and eosinophils, as well as by cells in the epithelium, fibroblasts, and smooth muscle cells [11-13]. These cells express three isoforms of TGF-β, namely TGF-β1, TGF-β2, and TGF-β3, with TGF-β1 being the most abundant [14]. Though TGF-β1 has both pro- and anti-inflammatory effects [11-13], studies have shown this cytokine to primarily suppress inflammation and promote healing following tissue injury in the upper urinary tract [14,15]. The numerous biological functions of all TGF-β's require an initial bioactivation, in which the dimeric TGF-β precursor is cleaved intracellularly to yield the active TGF-β dimer, which subsequently remains associated with the remaining portion of its own pro-form, the latency-associated peptide (LAP). This latent TGF-β complex is secreted, and may bind to other proteins such as latent TGF-β binding proteins (LTBP) or α2-macroglobulin [16,17]. Bioactive TGF-β1 is a potent suppressor of iNOS expression and enzymatic activity [18].

The excessive production of TGF-β1 can promote tissue fibrosis in a number of diseases including liver cirrhosis, pulmonary fibrosis, and fibrotic kidney [19]. Coincidentally, a significant degree of fibrosis is also frequently noticed in the bladder of chronic IC patients on cystoscopic exam, the reasons for which remain elusive [20-22]. Experimental IC is also induced in rats by acrolein, a metabolite of CYP excreted into the urine from the kidney [3]. This animal model exhibits gender-based differences in the observed pathology [23-25], a feature also seen in human IC [26]. A study on ovariectomized rats revealed an increased severity of histological changes induced by CYP that were ameliorated by estrogen replacement [25]. A similar gender disparity in human lower urinary tract diseases is exemplified by significantly higher levels of IL-1α and IL-1RA in urine of healthy females that seem to provide prophylaxis against upper and lower urinary tract infection [26]. Steroid hormones released from the ovary can induce expression of IL-1RA and slow down the progression of renal diseases [27].

We hypothesized that urine levels of TGF-β1 are not specific for nephropathy, but can also reflect the state of the acrolein-injured bladder. Given the interplay of regulatory influences operating in the production of NO, TGF-β1 and other pro-inflammatory cytokines in bladder inflammation, we sought to define the time-dependent changes in the urinary levels of NO-derived oxidation products as well as TGF-β1 in a rat model of CYP-induced cystitis. We also sought to determine if there are gender-specific patterns of iNOS and TGF-β1 expression in this animal model. We further sought to determine the expression and cellular localization of active and latent TGF-β1 as well as that of iNOS in the bladder. Our findings demonstrate lower levels of iNOS and NO reaction products, and concomitantly higher levels of TGF-β1, in male vs. female rats. We discuss the possible relevance of these findings to the pathology and possible diagnosis and treatment of human IC.

## Methods

All animal experimentation described was performed in accordance with NIH guidelines following approval by the University of Pittsburgh Institutional Animal Care and Use Committee (IACUC). Cyclophosphamide was procured from Sigma-Aldrich (St. Louis, MO). Intraperitoneal CYP injections [28] were performed in 4-month old Sprague-Dawley rats of either sex. Urine specimens obtained from rats kept in metabolic cages during daylight hours were frozen immediately in liquid nitrogen and stored at -80°C prior to analysis. Baseline urine samples were obtained throughout the 12 daylight hours prior to next day's CYP injection, as well as from vehicle-treated rats. Bladder tissue was harvested from both CYP- and vehicle-treated rats. Harvested bladders were split into two halves. One half was cryopreserved for immunohistochemistry and the other half was frozen immediately for protein analysis.

### Measurement of NO reaction products and TGF-β1

Frozen urine samples from each hourly interval were thawed, and 20 μl of each sample were analyzed. NO was measured as NO_2_^-^/NO_3_^- ^by the nitrate reductase method [29] using a commercially available kit (Cayman Chemical, Ann Arbor, MI) according to manufacturer's protocol. Fifty μL from each sample were analyzed for active and latent TGF-β1 in triplicate using a commercial antigen capture ELISA kit (Quantikine™, R&D Systems, Minneapolis, MN). Each sample was assayed both in the absence and presence of 1 M HCl in order to assess both active and latent TGF-β1, respectively. Urine levels of NO_2_^-^/NO_3_^- ^and TGF-β1 were normalized to the respective creatinine concentrations and expressed as μmol per mg of creatinine and pg/mg of creatinine, respectively. At the conclusion of the study, harvested bladders were homogenized, lysed, and stored at -80°C. All tissue TGF-β1 values were then standardized by bladder weight and expressed as μg per bladder.

### Immunostaining and Confocal Microscopy of iNOS, active TGF-β1, and latent TGF-β1

Bladders were fixed in formalin and frozen with TBS tissue freezing medium (Pacific Southwest Lab Equipment Inc., CA) prior to sectioning to a sample thickness of 8 microns. Tissue was permeabilized with 0.2% Triton X-100-PBS for 15 min, followed by a 1 h block in 2% BSA-PBS. Tissue sections were incubated in 0.5% BSA-PBS with 5 μg/ml of chicken-anti-TGFβ1 (to assess the expression of active TGF-β1) and goat-anti human LAP (to assess total/latent TGF-β1) [30]. Both antibodies were obtained from R&D Systems. Mouse anti-human iNOS antibody was obtained from Santa Cruz Biotechnology (Santa Cruz, CA) and used at a concentration of 2 μg/ml. The primary antibodies were incubated overnight at 4°C (anti-TGF-β1 and anti-LAP) or at room temperature for 1 h (anti-iNOS). Anti-LAP antibody was used for the immunodetection of latent TGF-β1. Following primary antibody incubation, the sections were washed 3× with 0.5% BSA-PBS and incubated with the appropriate secondary antibodies in 0.5% BSA-PBS for 1 h at room temperature. Secondary antibodies were as follows: donkey-anti-chicken Cy3 (1:1000, Jackson ImmunoResearch, West Grove, PA), donkey-anti-goat Cy5 (1:500, Jackson ImmunoResearch), donkey-anti mouse Alexa488 (1:500, Invitrogen), Alexa488-phalloidin (1:250, Invitrogen, Carlsbad, CA), or Alexa647-phalloidin (1:250, Invitrogen). The tissue sections were then washed 3× with 0.5% BSA-PBS, followed by 3× washes with PBS. Nuclei were stained for 10 s with Hoechst dye (1 mg/100 ml bisbenzimide). The slides were rinsed with PBS and coverslipped with Gelvatol, a water-soluble mounting media (a mix of 21 g polyvinyl alcohol in 42 ml glycerol, 52 ml water, a few crystals of sodium azide, and 106 ml 0.2-M Tris buffer, pH = 8.5). The slides were then visualized with a confocal microscope (Fluoview 1000; Olympus, Melville, NY).

### Statistical Analysis

Values are expressed as mean ± SEM. Analysis of parametric data among experimental groups of different sex at baseline and after CYP injection was carried using one way ANOVA followed by Tukey's multiple comparison tests for statistical significance. The Pearson correlation coefficient using two tailed test for significance was used to check inverse correlation. Significance was considered at p < 0.05.

## Results

### Micturition at Baseline and After CYP

#### Baseline assessment

**Figure 1:**
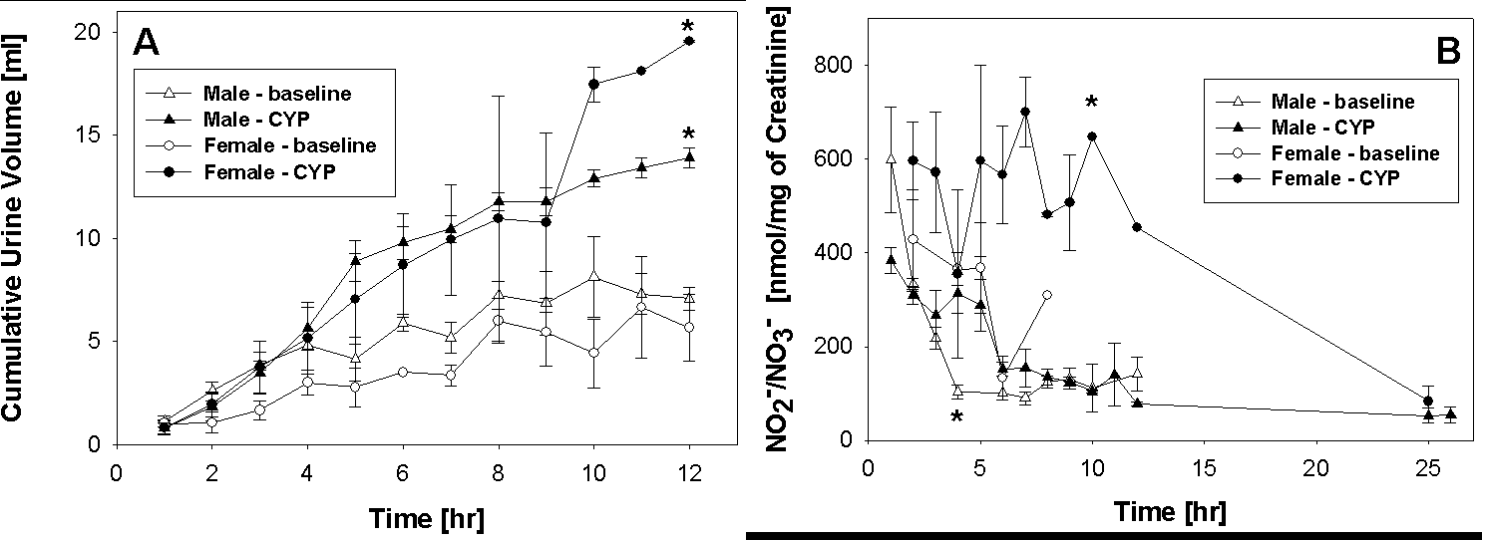
**Urinary profile and baseline levels of NO reaction products**. Panel A:- Effect of CYP on micturition pattern. Cumulative urine volume was measured over the period of 12 daylight hours before and after CYP injection (150 mg/kg) in male and female rats. In absence of CYP, female rats (empty black dot) voided a cumulative volume of 5.88 ± 1.88 ml compared to slightly higher volume of 7.67 ± 0.59 ml in male rats (empty black triangle). The mean urinary frequency was 7.8 ± 0.54 in female rats and 7.57 ± 0.86 in male rats during the 12-h time period at baseline. The cumulative urine volume increased significantly to 9.65 ± 2.34 ml in female (solid black dot) and to 12.9 ± 1.03 ml in male rats (solid black triangle) after CYP, relative to baseline values in female rats (ANOVA, Tukey post hoc test; *p < 0.05). The mean urinary frequency also increased significantly after CYP to 19 ± 1.5 and 20.25 ± 2.6 in males and females, respectively. Panel B - Urine levels of NO reaction products at baseline and after CYP. NO_2_^-^/NO_3_^- ^are expressed as μmol/mg creatinine. The measurement of NO_2_^-^/NO_3_^- ^in individual urine voids from control male rats showed that levels of NO reaction products do not remain constant throughout the day, but are maximal at the beginning of day and then stabilize for the remainder of the day. Values at baseline in female rats (empty black dot) did not change over the course of the day. The levels of NO products in urine of CYP treated female rats (solid black dot) were significantly higher compared to male rats at baseline (empty black triangle) and after CYP injection (solid black triangle) (ANOVA, Tukey post hoc test; *p < 0.01).

Cumulative urine volume for a 12-h period a day prior to CYP injection and on the day of injection was measured and plotted (Fig. 1A). At baseline, male rats showed a slightly higher cumulative urine volume (7.67 ± 0.59 ml) than female rats (5.88 ± 1.88 ml), but the differences were not statistically significant (ANOVA, Tukey's Multiple Comparison post-test; p > 0.05; n = 8 rats per group). Both female and male rats voided urine with similar average frequency at baseline (Fig. 1A), as measured by the number of urination events in a single 12-h period (7.8 ± 0.54 for females and 7.57 ± 0.86 for males; n = 8 rats per group).

### Assessment following treatment with CYP

As previously reported by our group, characteristic dysfunctional voiding after CYP injection (150 mg/kg) [4] in female rats was also observed in male rats. The cumulative urine volume voided as well the urination frequency in rats of both genders drastically increased for the same 12-h period. The cumulative urine volume increased to 9.65 ± 2.34 ml in females and 12.9 ± 1.03 ml in males (Fig. 1A). The rise in cumulative urine volume in female and male rats after treatment with CYP was significant relative to baseline values only in female rats (ANOVA, Tukey post test comparison; *p < 0.05, n = 4 rats per group). The average 12-h frequency in male rats after CYP was 19 ± 1.5 vs. 20.25 ± 2.6 (n = 4) in females (not statistically significant).

In addition, urinary frequency, as measured by urination events for each hour, showed a dramatic increase during the time period of 4-8 h after CYP injection. Female rats urinated on an average of five times per hour compared to three times per hour in male rats during this time period. These results corroborate the previously-reported high urination frequency after CYP relative to baseline [4]. Occasional microhematuria was also noted in few of the urine specimens from this time period (data not shown).

### Urinary Levels of NO Reaction Products at Baseline and After CYP Injection

#### Baseline assessment

Urine levels of the NO oxidation products NO_2_^-^/NO_3_^- ^served as a proxy for the magnitude of NO production in bladder tissue. The maxima and minima of NO_2_^-^/NO_3_^- ^during the day in control rats were reciprocal to the maxima and minima of total TGF-β1 at baseline in both sexes (Pearson correlation coefficient = 0.2 [two tailed p = 0.56; n = 4] for males and 0.19 [p = 0.75; n = 4] for females; Fig. 1B).

#### Assessment following treatment with CYP

Our results demonstrated an elevation of NO reaction products in the urine of CYP-treated rats when compared to the levels observed in control rats collected at the same time point of the day. The levels of NO_2_^-^/NO_3_^- ^in the urine of CYP-treated female rats remained higher as compared to both CYP-treated and control male rats (ANOVA, Tukey post test comparison; *p < 0.01, n = 4 rats per group). Female rats showed the highest levels of NO_2_^-^/NO_3_^- ^6 h post-CYP, followed by a steady decline to levels lower than baseline at 24 h. Treatment of male rats with CYP was associated with a sharp rise in levels of NO_2_^-^/NO_3_^- ^at 4 h that remained elevated until 6 h and then progressively declined to lower values (Fig. 1B).

### Levels of TGF-β1 in Urine

#### Baseline assessment

**Figure 2:**
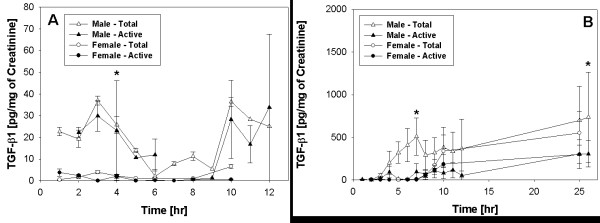
**Urine levels of active and latent/total TGF-β1 at baseline and after CYP**. Active and latent/total TGF-β1 values are reported as pg/mg of creatinine. Panel A - Urine levels of TGF-β1 at baseline. In the absence of CYP injection, male and female rats excreted very low amounts of TGF-β1. Total (**empty black triangle**) and active (**solid black triangle**) forms of TGF-β1 in male urine were significantly higher than total (**empty black dot**) and active (**solid black dot**) forms in female urine p < 0.001 (n = 8). The TGF-β1 levels in male urine were at least 10-fold higher than levels in female urine. Panel B - Urine levels of TGF-β-1 after CYP. Injection of CYP induced time dependent 100-fold increase in urine levels of TGF-β1 in rats of both sexes relative to the respective baseline values. TGF-β1 levels after CYP were significantly higher than respective baseline values only in male urine and not in female urine (ANOVA, Tukey's *post hoc *test; p < 0.01). The levels of total TGF-β1 (**empty black triangle**) in male urine after CYP were also significantly higher than the levels of active (**solid black dot**) and total (**empty black dot**) TGF-β1 in female urine both at baseline and after CYP (ANOVA, Tukey *post hoc *test; p < 0.01). The urine levels of total TGF-β1 (empty black triangle) were significantly higher than those of active TGF-β1 (solid black triangle) only in male urine and not in female urine after CYP (*p < 0.01).

Urine analysis of male (Δ) and female (•) rats before CYP injection revealed secretion of TGF-β1 in very low amounts (Fig. 2A). The levels of latent/total and active forms of TGF-β1 in males were significantly higher than the respective forms of TGF-β1 in females (*p < 0.001; n = 8). There was positive correlation between active and latent forms of TGF-β1 in urine with Pearson's coefficient of 0.98 (two tailed *p < 0.0001) and 0.87 (two tailed *p < 0.0001) for female and male rats, respectively. The levels of active and total TGF-β1 were maintained at similar levels throughout the day in male rats.

#### Assessment following treatment with CYP

A progressive rise of TGF-β1 was observed in the urine of male and female rats after CYP injection, starting at 5 h (Fig. 2B). TGF-β1 levels continued to rise over the 12-h period of urine collection, reaching a maximum when experiment was terminated at 24 h. The urine levels of total TGF-β1 in rats of both sexes rose nearly 100-fold at 24 h relative to their respective baseline values (Fig. 2B), though this change was significantly higher vs. baseline values only in male urine (ANOVA, Tukey's Multiple Comparison post-test; p < 0.01). The levels of total/latent TGF-β1 in the urine of male rats after CYP were also significantly higher than the levels of active and total TGF-β1 in the urine of female rats, both at baseline and after CYP (ANOVA, Tukey post test comparison; p < 0.01).

### Correlation for Urine levels of TGF-β1 and NO_2_^-^/NO_3_^-^

**Figure 3:**
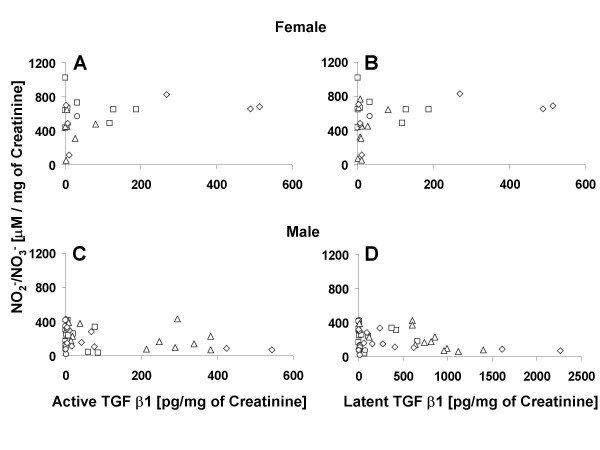
**Inverse Relationship between urine TGF-β1 and NO_2_^-^/NO_3_^- ^levels**. Dot matrix plot of NO_2_^-^/NO_3_^- ^in relation to active and total TGF-β1 in urine of female (Panel A & B) and male (Panel C & D) rats. The different dots (circle, triangle, square and diamond) represent values of individual rats of each sex at different time points. Mean urinary levels of NO_2_^-^/NO_3_^- ^in female rats were inversely correlated to mean total TGF-β1 (Panel B) and active TGF-β1 (Panel A), with Pearson correlation coefficients of -0.72 (two tailed; *p < 0.02) and -0.69 (two tailed; *p < 0.03), respectively. Mean urine levels of NO_2_^-^/NO_3_^- ^in male rats were inversely correlated to mean total TGF-β1 (Panel D) and active TGF-β1 (Panel C), with Pearson correlation coefficients of -0.89 (two tailed; *p < 0.0001) and -0.76 (two tailed; *p < 0.01), respectively.

The urinary levels of NO metabolites NO_2_^-^/NO_3_^- ^were inversely correlated to active TGF-β1 and latent TGF-β1 in both male and female rats (Fig. 3). The Pearson correlation coefficient in female rats was -0.69 (two tailed; *p < 0.03) and -0.72 (two tailed; *p < 0.02) for relationship of NO_2_^-^/NO_3_^-^, with active TGF-β1 (Fig. 3A) and latent TGF-β1 (Fig. 3B), respectively. In male rats, the Pearson correlation coefficient was -0.89 (two tailed; *p < 0.0001) and -0.76 (two tailed; *p < 0.01) for latent TGF-β1 (Fig. 3D) and active TGF-β1 (Fig. 3C), respectively.

### Levels of TGF-β1 in Bladder Tissue following CYP injection

**Figure 4:**
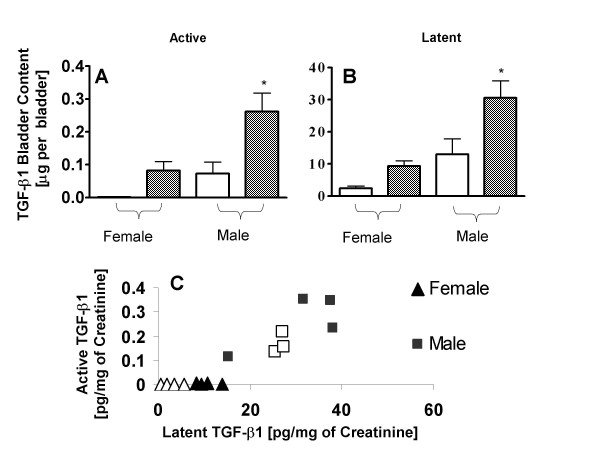
**Bladder tissue levels of TGF-β-1 in control and CYP-treated rats**. Bladder lysate from different groups were analyzed for TGF-β1 by ELISA, and levels of TGF-β1 were then standardized by bladder weight and expressed as μg per bladder. Male rats exhibited the highest expression of TGF-β1 in tissue compared to tissue levels of other groups (ANOVA, Tukey's Multiple post hoc test; *p < 0.05). Levels of active TGF-β1 (Panel A) in bladder tissue were nearly 100-fold higher than levels of latent TGF-β1 (Panel B) measured in bladder tissue of all groups. The substantial presence of latent TGF-β1 in female rats at baseline (open bars) and after CYP (shaded bars) was accompanied by only a minor presence of active TGF-β1 (0 - 3.6 ng; Panel C). In contrast, male rats exhibited both active and latent TGF-β1 at baseline and after CYP, with positive Pearson's coefficients of 0.65 and 0.75, respectively but without statistical significance (p = 0.24).

We sought to determine if the gender-associated differences in urinary TGF-β1 levels stemmed from differences in expression of TGF-β1 in the bladder. Similar to what was found in urine, female rats at baseline had the lower levels of both total and active TGF-β1 in bladder tissue as compared to their male counterparts (Fig. 4). Higher levels of TGF-β1 in urine of male rats were associated with significantly higher levels of this cytokine in bladder tissue as compared to the tissue levels in the other experimental groups (ANOVA, Tukey post test comparison; *p < 0.05; Fig. 4). The 100-fold difference in the magnitude of tissue levels for latent TGF-β1 (panel B) and active TGF-β1 (panel A) was maintained across all groups. The substantial levels of latent TGF-β1 in female rats at baseline and after CYP was accompanied by only minor levels of active TGF-β1 in bladder tissue (0 - 3.6 ng; Fig. 4C). In contrast, male rats exhibited substantial levels of both active and latent TGF-β1 at baseline and following treatment with CYP, with positive Pearson's coefficients of 0.65 and 0.75, respectively (p = 0.24; Fig. 4C).

### Immunocytochemical Localization of TGF-β1 and iNOS

**Figure 5:**
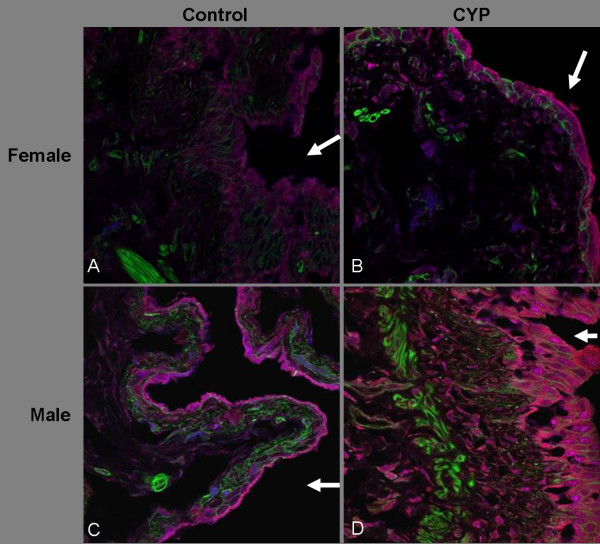
**Localization of TGF-β1 in rat bladder**. Control and CYP-treated bladders were harvested at 24 h after CYP injection, fixed in formalin, and cryopreserved prior to sectioning to a thickness of 8 μm. Bladder sections were stained for TGF-β1 (red fluorescence) and LAP (blue fluorescence) for the immunodetection of active and latent TGF-β1, respectively. The urothelium region of sections was marked by a lower degree of green stain for smooth muscle actin/phalloidin. Male CYP-treated rats exhibited the most intense magenta stain to indicate the substantial presence of active TGF-β1 in urothelium (Panel D; *lumen marked by white arrow*), that was much lower in control male rats (Panel C) and nearly absent in female control rats (Panel A). The purple color emerging from the predominance of blue fluorescence in the overlap with red fluorescence was more prominent in controls of both genders as well as in female rats treated with CYP, but absent in male CYP-treated rats. Magnification is 60× in all sections and is representative of 4 animals in each group. The experiment is representative of 5 fields per slide.

Having established the presence of gender based differences in NO_2_^-^/NO_3_^- ^and latent TGF-β1 levels in urine from control and CYP-treated animals, we next sought to detect protein expression and localization of iNOS as well as active and latent TGF-β1. Accordingly, bladders from control and CYP-treated animals were harvested at 24 h from the initiation of the experiment, fixed in formalin, and subjected to immunocytochemistry for iNOS as well as active and latent TGF-β1 followed by confocal microscopy. In bladder tissue sections, active TGF-β1 is represented by red fluorescence and latent/total TGF-β1 (visualized by immunostaining for LAP) is represented by blue fluorescence, while green stain represents smooth muscle actin/phalloidin (Fig. 5). The urothelium region of sections was marked by a lower expression of actin/phalloidin. The purple color in the panels (Fig. 5A-C) indicates the predominance of blue fluorescence of latent TGF-β1 over the red fluorescence of active TGF-β1. The magenta color (Fig. 5D) in the panels indicates overlap of similar intensity of blue fluorescence from latent TGF-β1 and the red fluorescence of active TGF-β1. In agreement with the ELISA results in bladder tissue, male CYP-treated rats exhibited the most intense magenta stain as compared to other groups, indicating higher expression of active TGF-β1 in the urothelium (Fig. 5D; *lumen marked by white arrow*). The expression of active TGF-β1 was much lower in control male rats (Fig. 5C) and female control rats (Fig. 5A). The purple color is more evident in controls of both sexes and in female CYP-treated rats (Figs. 5A-B), suggesting that latent TGF-β1 was elevated and activated to a moderate degree in these tissues.

**Figure 6:**
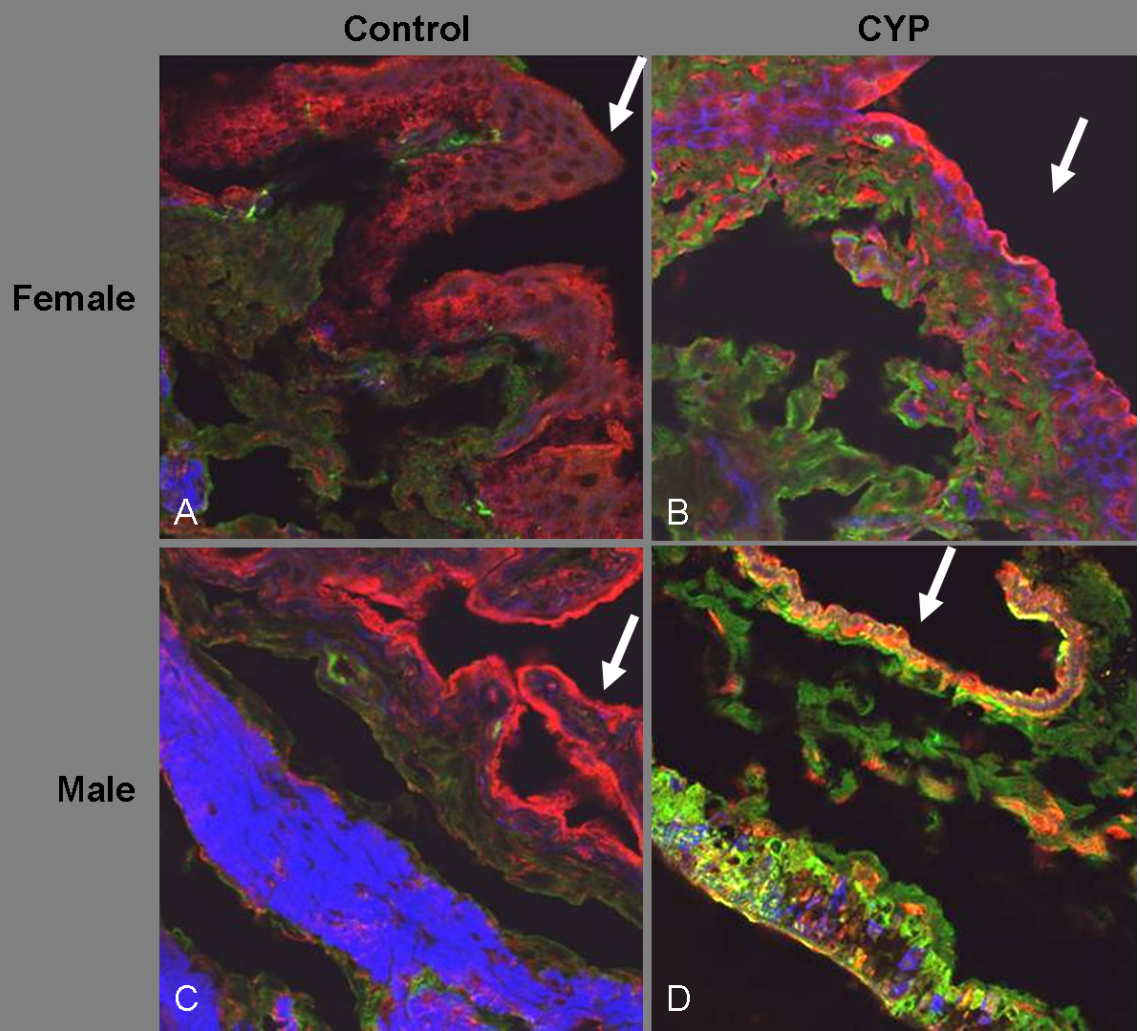
**Co-localization of TGF-β1 and iNOS in rat bladder**. The confocal images show iNOS (red stain) and active TGF-β1 (green stain) in bladder sections. iNOS appears to be expressed at low levels in control male (Panel C) and female (Panel A) rats. In contrast, iNOS immunostaining is increased following treatment with CYP (Panels B and D). The red stain for TGF-β1 was mostly localized in the urothelium region of all the groups. This region was also marked by a lower degree of blue stain for smooth muscle actin/phalloidin in all the groups. Tissue destruction caused by CYP is prominent in Panel B and D relative to the normal tissue architecture observed in Panels A and C. The lumen region adjoining the urothelium is indicated by white arrows. Magnification is 60× in all sections. The experiment is representative of 5 fields per slide.

We next sought to determine if our emerging impression of reciprocal expression of iNOS and TGF-β1 in the setting of CYP-induced bladder inflammation could be confirmed immunocytochemically at the cellular level. In Fig. 6, iNOS is visualized in green and active TGF-β1 is red. Except for male CYP-treated rats, the urothelium of other groups was distinctly red and cells below the lumen were stained green, indicating predominant iNOS expression and low active TGF-β1. The male rats showed regions of equal intensity for red and green fluorescence, just below the cell layer bordering the lumen. Accordingly, we conclude that both iNOS and active TGF-β1 are expressed in this region, though not co-expressed in the same cells. This narrowing of tissue regions expressing green and red fluorescence probably results from more severe tissue destruction induced by acrolein from CYP in male rats relative to other groups.

Immunocytochemistry corroborated the urine and tissue levels of TGF-β1 and NO_2_^-^/NO_3_^-^. In support of the tissue ELISA data, bladder tissue from female CYP-treated rats (Fig. 6B) exhibited the most intense green stain for iNOS in the urothelium as compared to the other groups. The immunocytochemical expression of iNOS was much lower in control male rats (Fig. 6C). The bladders of female control rats (Fig. 6A) exhibited an elevated expression of iNOS relative to control male rats.

## Discussion

A central observation of our study was the *in vivo *evidence for inverse relationship between TGF-β1 and iNOS/NO synthesis in the setting of bladder inflammation: when NO_2_^-^/NO_3_^- ^were at their lowest (24 h after CYP injection), urinary TGF-β1 level reached their peak in both male and female rats. These results suggest that TGF-β1 is an endogenous negative regulator of iNOS and subsequent production of NO reaction products, a notion supported in several biological settings [31-37]. Further support for this hypothesis comes from our immunostaining studies showing reciprocal staining of iNOS and TGF-β1, studies that agree with previous reports on TGF-β1 synthesis by epithelial and immune cells [12]. Those studies, along with ours, suggest that the urothelium is the likely source of TGF-β1 and NO metabolites measured in urine [12]. Prior studies in rat smooth muscle cells suggested that bioactive TGF-β1 is a potent suppressor of iNOS expression and enzymatic activity [18]. Further studies in this cell type have also shown that TGF-β1 does not directly inhibit enzymatic activity of iNOS, but rather that this cytokine both suppresses the induction of iNOS mRNA as well as increases the degradation of iNOS protein [38,39].

In the present study, we also observed a constitutive, basal secretion of active TGF-β1 in the urine. The levels of active TGF-β1 in the urine were generally correlated with the levels of latent/total TGF-β1 in the urine of both males and females (with the possible exception of the 10-h time point in untreated male rats), suggesting that there is an elevation in the expression of total TGF-β1 and that a constant fraction of total TGF-β1 is active in urine regardless of whether or not the animals were exposed to CYP. We hypothesize that this active TGF-β1 originates in the bladder urothelium due to our data on the presence of TGF-β1 in the bladders of control rats. Likewise, the expression of iNOS is observed to a low degree in control rat bladder.

The basal secretion of TGF-β1 and NO_2_^-^/NO_3_^- ^seems to fluctuate slightly throughout the day. Interestingly, we noted that the maxima and minima of urinary TGF-β1 and NO_2_^-^/NO_3_^- ^occurred at reciprocal time points of each other, supporting the hypothesis that TGF-β1 is a physiological suppressor of iNOS. The levels of NO_2_^-^/NO_3_^- ^do not remain constant throughout the day, but progressively fall from maximum levels measured during the morning hours. The rise of NO_2_^-^/NO_3_^- ^in the morning before falling to a stable value suggests that some of the NO_2_^-^/NO_3_^- ^assessed are contributed through the enzymatic action of the constitutive NOS enzymes (endothelial NOS and neuronal NOS) as well as potentially from the stress to the animal from handling and transport to metabolic cage from animal facility. These results may indicate an interplay among components of the endocrine system, especially the hypothalamic-pituitary-adrenal (HPA) axis that regulates circadian rhythm and stress response with paracrine signaling in the bladder, a phenomenon previously demonstrated in the aorta [40]. Indeed, given that activation of TGF-β1 is increased in settings of physiological stress, our data may suggest involvement of TGF-β1 in the homeostatic mechanism linked to HPA axis [41]. It is worth noting that this large diurnal variation in urinary levels of NO reaction products and TGF-β1 in control rats argues for the need to sample urine at multiple time points in studies assessing inflammatory analytes in urine.

In contrast to earlier studies, we now demonstrate interrelated NO_2_^-^/NO_3_^- ^and TGF-β1 levels in individual urine voids separated by as little as 5 min in CYP-treated rats. Our results show elevation of urinary NO reaction products in CYP-treated rats when compared to control rats in urine collected at the same time point of the day. The peak levels of NO metabolites in urine occurred at 4 h post-CYP for male rats and at 6 h post-CYP for female rats relative to baseline values, and the increase at these time points agree with results reported previously [5]. It is not clear at this point why the peak level of urinary NO_2_^-^/NO_3_^- ^was delayed in females vs. males, but this phenomenon may be related to the influence of HPA axis and ovarian hormones on the expression of TGF-β1 in the bladder. It should be noted, however, that a previous study reported that the overall effects of estrous stage on CYP-induced bladder inflammation were insignificant [24]. In order to fully address this issue, the effect of cyclical changes in ovarian hormones will likely have to be determined by repeating the experiments described here in ovariectomized rats [42,43].

Our prior studies showed that other cytokines reach their peak by 4 h and decline by 24 h in the acute CYP model [4]. In contrast, the levels of TGF-β1 were negligible by 4 h, with peaks at 24 h consistent with a late, anti-inflammatory, and pro-healing role for this cytokine demonstrated in bronchial epithelial cells [12]. Indeed, it is known that inflammation induced by CYP begins to resolve by 24-48 h, and studies in other organs have confirmed the role of TGF-β1 in wound healing after injury and as regulator of immune cell activation in response to inflammation [44-46].

The reciprocal relationship of TGF-β1 with NO reaction products, as well as with other pro-inflammatory cytokines [4] seems to suggest a need for different stimuli for TGF-β1 production by the bladder [13]. One likely stimulus for the generation of TGF-β1 in the bladder might be reactive oxygen species (ROS) generated by acrolein, which can alter the redox balance in bladder tissues and lead to the activation of latent TGF-β1 [2]. Activated TGF-β1 is known to either decrease or increase the generation of ROS, depending on cellular/enzymatic source and experimental conditions [47-50] and therefore elevated levels of latent TGF-β1 in CYP-treated rats may also explain the reduced expression of TNF-α (a ROS-activated gene) in bladder noted by ourselves and others [4,51].

Our findings are likely to have clinical relevance. An elevated iNOS activity has been previously noted in IC patients, and elevated levels of NO reaction products have been linked to changes in tight junction protein dynamics associated with the observed disrupted barrier function of the urothelium [52]. In those studies, the release of TNF-α and IL-1β from bladder was shown to induce iNOS [53]. Our results demonstrating increased urinary NO_2_^-^/NO_3_^- ^after treatment with CYP agree with previous reports that assessed NO_2_^-^/NO_3_^- ^in urine collected over a 2-h time period from 2-4 h and 4 to 6 h after CYP injection [5].

The presence of urine TGF-β1 has not been previously described in IC patients, though enhanced expression of this cytokine has been noted in tissue biopsies of IC patients [9,10]. However, concurrent presence of iNOS and TGF-β1 in IC patients remains to be studied. Given the known biology of TGF-β1 and the elevated levels of this cytokine measured in our rat model, it is tempting to speculate that the bladder fibrosis characteristic of IC patients [20,54] may be caused at least in part by TGF-β1. In support of this hypothesis, incubation of human detrusor smooth muscle cells with TGF-β1 led to hypertrophic and fibrotic responses characterized by the upregulation of COL1A1 and COL3A1 mRNA; genes that are necessary for collagen synthesis [55].

The striking, gender-specific pattern of expression and secretion of TGF-β1 may be related to that seen in prior studies documenting hypertrophied lamina propria and stromal hyperplasia only in male mice, lacking type II TGF-β1 receptor gene [56]. The higher female prevalence of IC, as well as the limited clinical success in the treatment of hemorrhagic cystitis with estrogens [57,58], support the notion that TGF-β1 is a central molecular mediator governing the gender-related differences in the response to CYP reported here. Estrogen can reverse the effects of TGF-β1 by reducing the activity of the transcription factors Sp1 and Smad3, which in turn leads to the reduced synthesis of collagen and extracellular matrix [59,60]. A recent report reviewed five case studies of successful treatment of hemorrhagic cystitis with conjugated estrogens in the clinic, and showed that this therapeutic benefit was accompanied by an altered serum cytokine profile [58].

## Conclusion

The results of this study suggest that there exists an inverse relationship between the expression of TGF-β1 and NO reaction products in the acrolein-inflamed bladder. The inverse correlation between urine levels of NO-derived products and TGF-β1 may be viewed as a consequence of a more predominant TGF-β1 effect in blocking iNOS induction. Given the time course of inflammation induced by CYP in our study, TGF-β1 is likely to emerge as a central mediator of the resolution of inflammation and induction of healing and in the bladder. Our results therefore argue in favor of evaluating urinary TGF-β1 in IC patients in order to assess disease progression, and may point to novel areas for the development of therapeutics for this disease.

## Competing interests

The authors declare that they have no competing interests.

## Authors' contributions

PT and YV designed the study. DB, PL, VT and EW executed the experiments in the manuscripts. NY and RZ were involved in data analysis and manuscript preparation. All authors read and approved the final manuscript.

## References

[B1] Hu RQ, Mehter H, Nadasdy T, Satoskar A, Spetie DN, Rovin BH, Hebert L (2008). Severe hemorrhagic cystitis associated with prolonged oral cyclophosphamide therapy: case report and literature review. Rheumatol Int.

[B2] Korkmaz A, Topal T, Oter S (2007). Pathophysiological aspects of cyclophosphamide and ifosfamide induced hemorrhagic cystitis; implication of reactive oxygen and nitrogen species as well as PARP activation. Cell Biol Toxicol.

[B3] Cox PJ (1979). Cyclophosphamide cystitis - identification of acrolein as the causative agent. Biochem Pharmacol.

[B4] Smaldone MC, Vodovotz Y, Tyagi V, Barclay D, Philips BJ, Yoshimura N, Chancellor MB, Tyagi P (2009). Multiplex analysis of urinary cytokine levels in rat model of cyclophosphamide-induced cystitis. Urology.

[B5] Linares-Fernandez BE, Alfieri AB (2007). Cyclophosphamide induced cystitis: role of nitric oxide synthase, cyclooxygenase-1 and 2, and NK(1) receptors. J Urol.

[B6] Hamby ME, Hewett JA, Hewett SJ (2008). TGF-beta1 reduces the heterogeneity of astrocytic cyclooxygenase-2 and nitric oxide synthase-2 gene expression in a stimulus-independent manner. Prostaglandins Other Lipid Mediat.

[B7] Guo YS, Chen Z, Wen XD, Ko TC, Townsend CM, Hellmich MR (2008). Synergistic Regulation of COX-2 Expression by Bombesin and Transforming Growth Factor-beta. Dig Dis Sci.

[B8] Macedo FY, Baltazar F, Mourao LC, Almeida PR, Mota JM, Schmitt FC, Ribeiro RA (2008). Induction of COX-2 expression by acrolein in the rat model of hemorrhagic cystitis. Exp Toxicol Pathol.

[B9] Koskela LR, Thiel T, Ehren I, De Verdier PJ, Wiklund NP (2008). Localization and expression of inducible nitric oxide synthase in biopsies from patients with interstitial cystitis. J Urol.

[B10] Ueda T, Tamaki M, Ogawa O, Yoshimura N (2002). Over expression of platelet-derived endothelial cell growth factor/thymidine phosphorylase in patients with interstitial cystitis and bladder carcinoma. J Urol.

[B11] Chen ML, Yan BS, Bando Y, Kuchroo VK, Weiner HL (2008). Latency-Associated Peptide Identifies a Novel CD4+CD25+ Regulatory T Cell Subset with TGF{beta}-Mediated Function and Enhanced Suppression of Experimental Autoimmune Encephalomyelitis. J Immunol.

[B12] Mayer AK, Bartz H, Fey F, Schmidt LM, Dalpke AH (2008). Airway epithelial cells modify immune responses by inducing an anti-inflammatory microenvironment. Eur J Immunol.

[B13] Zheng SG, Wang J, Horwitz DA (2008). Cutting Edge: Foxp3+CD4+CD25+ Regulatory T Cells Induced by IL-2 and TGF-{beta} Are Resistant to Th17 Conversion by IL-6. J Immunol.

[B14] Bottinger EP (2007). TGF-beta in renal injury and disease. Semin Nephrol.

[B15] Saxena V, Lienesch DW, Zhou M, Bommireddy R, Azhar M, Doetschman T, Singh RR (2008). Dual roles of immunoregulatory cytokine TGF-beta in the pathogenesis of autoimmunity-mediated organ damage. J Immunol.

[B16] Annes JP, Munger JS, Rifkin DB (2003). Making sense of latent TGF beta activation. J Cell Sci.

[B17] Zamora R, Vodovotz Y (2005). Transforming growth factor-beta in critical illness. Crit Care Med.

[B18] Vodovotz Y (1997). Control of nitric oxide production by transforming growth factor-beta1: mechanistic insights and potential relevance to human disease. Nitric Oxide.

[B19] Gressner OA, Rizk MS, Kovalenko E, Weiskirchen R, Gressner AM (2008). Changing the pathogenetic roadmap of liver fibrosis? Where did it start; where will it go?. J Gastroenterol Hepatol.

[B20] Lemack GE, Zimmern PE (2001). [Interstitial cystitis: reevaluation of patients who do no respond to standard treatments]. Prog Urol.

[B21] MacDermott JP, Charpied GC, Tesluk H, Stone AR (1991). Can histological assessment predict the outcome in interstitial cystitis?. Br J Urol.

[B22] Lynes WL, Flynn SD, Shortliffe LD, Stamey TA (1990). The histology of interstitial cystitis. Am J Surg Pathol.

[B23] Rodo J, Farre X, Martin E (2001). Cyclophosphamide-induced hemorrhagic cystitis in rats that underwent colocystoplasty: experimental study. J Urol.

[B24] Bon K, Lanteri-Minet M, Menetrey D, Berkley KJ (1997). Sex, time-of-day and estrous variations in behavioral and bladder histological consequences of cyclophosphamide-induced cystitis in rats. Pain.

[B25] Terado M, Nomura M, Mineta K, Nishii H, Fujimoto N, Sasaguri T, Sasaguri Y, Matsumoto T (2005). Involvement of estrogen in the pathogenesis of cyclophosphamide-induced cystitis in rats. Endocrine.

[B26] Sadeghi M, Daniel V, Naujokat C, Weimer R, Opelz G (2005). Strikingly higher interleukin (IL)-1alpha, IL-1beta and soluble interleukin-1 receptor antagonist (sIL-1RA) but similar IL-2, sIL-2R, IL-3, IL-4, IL-6, sIL-6R, IL-10, tumour necrosis factor (TNF)-alpha, transforming growth factor (TGF)-beta and interferon IFN-gamma urine levels in healthy females compared to healthy males: protection against urinary tract injury?. Clin Exp Immunol.

[B27] Silbiger S, Neugarten J (2008). Gender and human chronic renal disease. Gend Med.

[B28] Vera PL, Wang X, Meyer-Siegler KL (2008). Upregulation of macrophage migration inhibitory factor (MIF) and CD74, receptor for MIF, in rat bladder during persistent cyclophosphamide-induced inflammation. Exp Biol Med (Maywood).

[B29] Vodovotz Y (1996). Modified microassay for serum nitrite and nitrate. Biotechniques.

[B30] Vodovotz Y, Chesler L, Chong H, Kim SJ, Simpson JT, DeGraff W, Cox GW, Roberts AB, Wink DA, Barcellos-Hoff MH (1999). Regulation of transforming growth factor beta1 by nitric oxide. Cancer Res.

[B31] Fang FC (1997). Perspectives series: host/pathogen interactions. Mechanisms of nitric oxide-related antimicrobial activity. J Clin Invest.

[B32] Olson MV, Lee J, Zhang F, Wang A, Dong Z (2006). Inducible nitric oxide synthase activity is essential for inhibition of prostatic tumor growth by interferon-beta gene therapy. Cancer Gene Ther.

[B33] Wang D, Lu S, Dong Z (2007). Regulation of TGF-beta1 gene transcription in human prostate cancer cells by nitric oxide. Prostate.

[B34] Shearer JD, Richards JR, Mills CD, Caldwell MD (1997). Differential regulation of macrophage arginine metabolism: a proposed role in wound healing. Am J Physiol.

[B35] Fiorenza G, Rateni L, Farroni MA, Bogue C, Dlugovitzky DG (2005). TNF-alpha, TGF-beta and NO relationship in sera from tuberculosis (TB) patients of different severity. Immunol Lett.

[B36] Lagadec P, Raynal S, Lieubeau B, Onier N, Arnould L, Saint-Giorgio V, Lawrence DA, Jeannin JF (1999). Evidence for control of nitric oxide synthesis by intracellular transforming growth factor-beta1 in tumor cells. Implications for tumor development. Am J Pathol.

[B37] Vodovotz Y, Zamora R, Lieber MJ, Luckhart S (2004). Cross-talk between nitric oxide and transforming growth factor-beta1 in malaria. Curr Mol Med.

[B38] Finder J, Stark WW, Nakayama DK, Geller D, Wasserloos K, Pitt BR, Davies P (1995). TGF-beta regulates production of NO in pulmonary artery smooth muscle cells by inhibiting expression of NOS. Am J Physiol.

[B39] Perrella MA, Jain MK, Lee ME (1998). Role of TGF-beta in vascular development and vascular reactivity. Miner Electrolyte Metab.

[B40] Navarro-Oliveira CM, Vassilieff VS, Cordellini S (2000). The sympathetic adrenomedullary system, but not the hypothalamic-pituitary-adrenal axis, participates in aorta adaptive response to stress: nitric oxide involvement. Auton Neurosci.

[B41] Smith EL, Batuman OA, Trost RC, Coplan JD, Rosenblum LA (2002). Transforming growth factor-beta 1 and cortisol in differentially reared primates. Brain Behav Immun.

[B42] Ewan KB, Shyamala G, Ravani SA, Tang Y, Akhurst R, Wakefield L, Barcellos-Hoff MH (2002). Latent transforming growth factor-beta activation in mammary gland: regulation by ovarian hormones affects ductal and alveolar proliferation. Am J Pathol.

[B43] Casslen B, Sandberg T, Gustavsson B, Willen R, Nilbert M (1998). Transforming growth factor beta1 in the human endometrium. Cyclic variation, increased expression by estradiol and progesterone, and regulation of plasminogen activators and plasminogen activator inhibitor-1. Biol Reprod.

[B44] Finch CE, Laping NJ, Morgan TE, Nichols NR, Pasinetti GM (1993). TGF-beta 1 is an organizer of responses to neurodegeneration. J Cell Biochem.

[B45] Ozawa H, Chancellor MB, Jung SY, Yokoyama T, Fraser MO, Yu Y, de Groat WC, Yoshimura N (1999). Effect of intravesical nitric oxide therapy on cyclophosphamide-induced cystitis. J Urol.

[B46] Welham NV, Lim X, Tateya I, Bless DM (2008). Inflammatory factor profiles one hour following vocal fold injury. Ann Otol Rhinol Laryngol.

[B47] Tsunawaki S, Sporn M, Ding A, Nathan C (1988). Deactivation of macrophages by transforming growth factor-beta. Nature.

[B48] Kim CD, Cho YJ, Park SH, Ha SW, Lee EG, Kim YJ, Kwon TH, Kim IS, Kim YL (2006). Urinary transforming growth factor-beta-induced gene-h3 (betaig-h3) as a sensitive predictor in chronic cyclosporine nephrotoxicity. Transplant Proc.

[B49] Hong YH, Peng HB, La Fata V, Liao JK (1997). Hydrogen peroxide-mediated transcriptional induction of macrophage colony-stimulating factor by TGF-beta1. J Immunol.

[B50] Islam KN, Kayanoki Y, Kaneto H, Suzuki K, Asahi M, Fujii J, Taniguchi N (1997). TGF-beta1 triggers oxidative modifications and enhances apoptosis in HIT cells through accumulation of reactive oxygen species by suppression of catalase and glutathione peroxidase. Free Radic Biol Med.

[B51] Helmy A, Hammam OA, El Lithy TR, El Deen Wishahi MM (2007). The role of TGF-beta-1 protein and TGF-beta-R-1 receptor in immune escape mechanism in bladder cancer. MedGenMed.

[B52] Hosseini A, Ehren I, Wiklund NP (2004). Nitric oxide as an objective marker for evaluation of treatment response in patients with classic interstitial cystitis. J Urol.

[B53] Ribeiro RA, Freitas HC, Campos MC, Santos CC, Figueiredo FC, Brito GA, Cunha FQ (2002). Tumor necrosis factor-alpha and interleukin-1beta mediate the production of nitric oxide involved in the pathogenesis of ifosfamide induced hemorrhagic cystitis in mice. J Urol.

[B54] Seishima M, Shimizu H, Oyama Z, Isogai K (2001). Mixed connective tissue disease following interstitial cystitis. Eur J Dermatol.

[B55] Howard PS, Kucich U, Coplen DE, He Y (2005). Transforming growth factor-beta1-induced hypertrophy and matrix expression in human bladder smooth muscle cells. Urology.

[B56] Sharif-Afshar AR, Donohoe JM, Pope JCt, Adams MC, Brock JW, Bhowmick NA (2005). Stromal hyperplasia in male bladders upon loss of transforming growth factor-beta signaling in fibroblasts. J Urol.

[B57] Nickel JC, Teichman JM, Gregoire M, Clark J, Downey J (2005). Prevalence, diagnosis, characterization, and treatment of prostatitis, interstitial cystitis, and epididymitis in outpatient urological practice: the Canadian PIE Study. Urology.

[B58] Kopterides P, Theodorakopoulou M, Mentzelopoulos S, Armaganidis A (2005). Cyclophosphamide-induced hemorrhagic cystitis successfully treated with conjugated estrogens. Am J Hematol.

[B59] Blush J, Lei J, Ju W, Silbiger S, Pullman J, Neugarten J (2004). Estradiol reverses renal injury in Alb/TGF-beta1 transgenic mice. Kidney Int.

[B60] Dixon A, Maric C (2007). 17beta-Estradiol attenuates diabetic kidney disease by regulating extracellular matrix and transforming growth factor-beta protein expression and signaling. Am J Physiol Renal Physiol.

